# Caries-Preventive Effect of a Public Health Programme for Pit and Fissure Sealant

**DOI:** 10.3290/j.ohpd.a44695

**Published:** 2020-07-04

**Authors:** Min Liu, Mei Zhao, Wei Chen, Qun Xu, Tao Peng

**Affiliations:** a Researcher and Executive Deputy Director, Department of Preventive Dentistry, Beijing Stomatological Hospital, Capital Medical University, Beijing, China; Researcher and Executive Deputy Director, Beijing Institute of Preventive Dentistry, Beijing, China. Study design, processing of data and analysis, primary preparation of manuscript.; b Researcher, Department of Preventive Dentistry, Beijing Stomatological Hospital, Capital Medical University, Beijing, China; Researcher, Beijing Institute of Preventive Dentistry, Beijing, China. Study design, data collection, processing of data and analysis, and preparation of the manuscript.; c Assistant Professor, Department of Preventive Dentistry, Beijing Stomatological Hospital, Capital Medical University, Beijing, China; Assistant Professor, Beijing Institute of Preventive Dentistry, Beijing, China. Study design, data collection, processing of data and analysis, and proofread the manuscript.; d Professor, Department of Epidemiology and Statistic, Peking Union Medical College, Beijing, China. Study design, data analysis, proofread the manuscript.; e Researcher, Department of Public Health, Shunyi District of Beijing Municipal Commission of Health, Beijing, China. Study design, data collection and preparation of the manuscript.

**Keywords:** caries, prevention, programme evaluation, sealant(s)

## Abstract

**Purpose:**

The caries-preventive effect of pit and fissure sealant was found to be related to the incidence of caries in the population. The rate of caries in China has been very low, and a pit and fissure sealant public health programme has been widely carried out since 2005. This study aims to evaluate the caries-preventive effect of this dental public health programme in Beijing, the capital of China.

**Materials and Methods:**

A 3-year longitudinal study was conducted from 2012 to 2015. All students (n = 2973) in one district of Beijing were included. Children who received a sealant were categorised into the sealant group (n = 1648), and the other children were categorised into the no sealant group (n = 1325).

**Results:**

The dental caries risk levels in the sealant group and the no sealant group were balanced at baseline. The caries incidences of children only counting four first molars after 28 months were 18.1% and 13.6% for the sealant group and the no sealant group, respectively (Chi-square test, p = 0.001). The risk ratio in the sealant group versus the no sealant group for caries yes/no (only four molars) at 28 months was 0.73 (95% CI, 0.60–0.90; p = 0.001), based on binary logistic regression.

**Conclusions:**

The pit and fissure sealant dental public health programme implemented in Beijing was effective in preventing dental caries in the first permanent molars.

Dental sealants were introduced in the 1960s to help prevent dental caries, mainly in the pits and fissures of occlusal tooth surfaces. Evidence suggests that fissure sealants are effective in preventing caries in children and adolescents compared to no sealants. Effectiveness may, however, be related to the caries incidence level in the population.^1^

China has a very low DMFT (decayed, missing, and filled teeth because of dental caries) index in children aged 12 years according to the figures presented in the WHO 2014 report^20^ (DMFT <1.2). The caries incidence level in China is increasing because of the growing consumption of sugars and inadequate preventive oral healthcare.^12,14,21^ According to the 3rd and 4th National Oral Health Epidemiology Surveys conducted in 2005 and 2015, respectively, caries prevalence in permanent teeth has increased from 28.9% to 38.5%, and the DMFT value has increased from 0.54 to 0.86 among 12-year-old children.^18^ A dental public health promotion programme for a pit and fissure sealant was first developed in Beijing, the capital of China, in 2005. This programme was financially supported by the government, and it was technically organised by the Beijing Institute of Preventive Dentistry (BIPD). Children aged 7–9 years were examined by dentists, and first permanent molars showing high caries risk indicators were sealed with resin-based sealants free of charge. More than 3 million permanent molars were sealed in Beijing from 2005 to 2016. The sealant programme was highly evaluated by the National Health Commission of the People’s Republic of China. Therefore, this sealant public health programme was accepted and applied in almost all of the provinces of China from 2008 to 2014. More than 4.3 million molars of 1.6 million children were sealed in 2015 in the whole country, and a total of 141 million RMB was spent (about 35 RMB per molar). The sealant programme was also highlighted in China’s long-term planning for the prevention and treatment of chronic diseases (2017–2025).

Considering the low caries risk background in permanent dentition in China, the caries-preventive effect of this programme was uncertain and had not been critically evaluated. The purpose of this study was to evaluate the caries-preventive effect of the dental public health programme of a pit and fissure sealant applied to the first permanent molars in Beijing, the capital of China.

## Materials and Methods

### Study Population

Beijing is a megacity with 16 districts. Shunyi District is located in the suburban area, and the fluoride concentration in tap water in this district is about 0.2–0.5 mg/L. A longitudinal study was conducted in 2016, which was authorised by the Beijing Municipal Health Commission and ethically approved by the Ethics Committee of Beijing Stomatological Hospital, Capital Medical University. In 2016, all primary school students from 38 schools, who were in the first semester of grade 6 and had received all four annual physical examinations from academic years 2012–2013 to 2015–2016, were included in the study population (n = 2973). The mandatory physical examination annually organised by the Health Center of Primary and Middle Schools should have covered all school children. The following items were included: body height, weight, eyesight, dental caries status, and so on. Dental caries in both the primary and permanent dentitions were examined by dental professionals according to the WHO 4th Basic Oral Health Survey Method developed in 1997.^19^ The DMFT index was used to evaluate the caries status of all the permanent teeth. DMFT (M4) was used to evaluate the caries status of only the four first permanent molars, and DMFT(–M4) was used to evaluate the caries status of all permanent teeth, except the four first permanent molars, in this study. The dental caries status was examined by dentists every year. All of the dentists had received standard training for the identification and recording of dental caries based on the WHO 4th Basic Oral Health Survey Method, but calibration tests were usually not performed. The annual physical examination was a routine assessment performed by the Health Center of Primary and Middle Schools, and it had been in place for more than 30 years.

Dental teams visited the primary schools and screened the children aged 7–9 years (mainly in Grades 2 to 3). The completely erupted first permanent molars showing high caries risk indicators were cleaned with a dental rotary brush, etched with 37% phosphoric acid using cotton rolls for moisture control, and then sealed by light-polymerised resin-based sealant. Sealants were applied to the teeth of only those children with written parental consent. Children were also allowed to visit dental clinics for service if they were not screened in their school or the schools were not covered by the programme. More than 90% of the sealants were applied in school with use of portable dental units, including mobile aspirators, and the other sealants were sealed in dental units. Sealants were checked 1 year later and were replaced if there was partial or total loss after the original application.

Indications for molars showing high caries risk indicators include the following^3–5^: a deep or irregular fissure and presence of a fossa or a pit, especially if it catches the tip of the explorer; an intact occlusal surface is present where the contralateral tooth surface is carious or restored; presence of stained pits and fissures with minimum decalcification or opacification and no softness at the base of the fissure is another criterion.

Both the occlusal and buccal/palatal surfaces were evaluated and sealed. Contraindications for sealant use include the following: a well-established cavitated caries lesion on any surface of the molar; presence of restoration, self-cleansing pits and fissures; and children who were not able to cooperate during the procedure.

**Fig 1:**
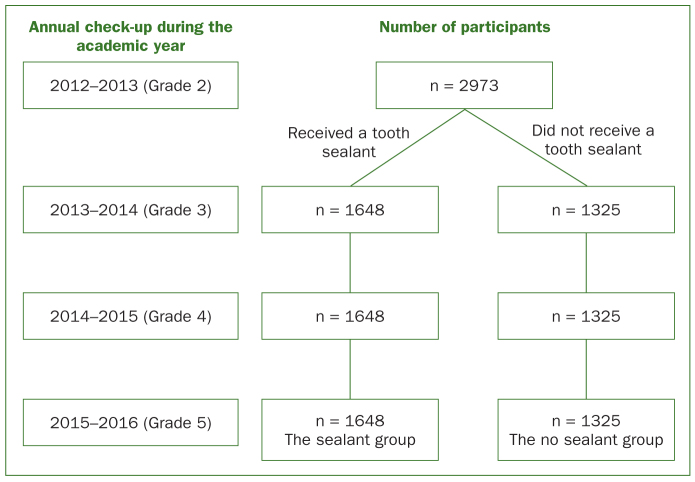
Flow diagram of the participants.

A total of 1648 students received a sealant (named the sealant group) between the two annual examinations performed during the academic years 2012–2013 and 2013–2014. The average review period (the time period from receiving the sealant to the last examination) was 28.2 months for these 1648 students, the number (SD) of sealed molars per child was 3.4 (0.9), and the age at which tooth sealing was performed (years, SD) was 8.7 years (0.7). The participants who did not receive any sealant were categorised into the no sealant group (n = 1325, Fig 1), which included three types of populations: schools or children who were not covered by this program, children of parents who did not sign the informed consent form, and very few children who did not meet the criteria. The accurate composition of each category was not available.

Data were processed and analysed by the Statistical Package for the Social Sciences (SPSS). The differences in proportions were evaluated by the Chi-square test, and differences in the mean values of the DMFT index between groups and various sections were tested by the non-parametric test. Binary logistic regression was used to identify the variables associated with dental caries. P <0.05 was considered to indicate statistical significance.

## Results

### The Comparative Test Between the Sealant Group and the No Sealant Group

The percentages of girls in the sealant group and the no sealant group at baseline were 46.0% and 47.8%, respectively (Chi-square test, p = 0.369). The prevalence of caries in primary teeth at baseline in the sealant group and the no sealant group were 62.6% and 65.1%, respectively (Chi-square test, p = 0.179), and the prevalence of caries in permanent teeth were 2.2% and 2.9%, respectively (Chi-square test, p = 0.197).

**Fig 2:**
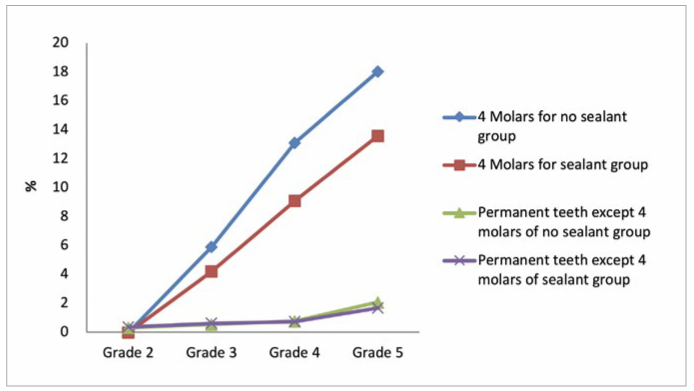
Incidences of caries in the sealant group and the no sealant group at the four annual check-up visits.

No statistically significant differences were found between the sealant group and the no sealant group with respect to age, body mass index (BMI), and caries incidences in the permanent teeth except the four first molars at the four annual check-up sections (Table 1 and Fig 2).

**Table 1:** Age, BMI and DMFT in the sealant group and the no sealant group at the four annual check-up visitations

Academic year	No sealant group	Sealant group	Total	Statistical test
	(n = 1325)	(n = 21648)	(n = 2973)	
Age (year, mean ± std )				Independent samples test
2012–2013	7.7 ± 0.5	7. 7 ± 0.4	7.7 ± 0.4	F = 5.775, p = 0.011
2013–2014	8.7 ± 0.4	8.7 ± 0.4	8.7 ± 0.4	F = 6.112, p = 0.051
2014–2015	9.8 ± 0.5	9.7 ± 0.4	9.8 ± 0.5	F = 7.692, p = 0.000
2015–2016	10.7 ± 0.4	10.7 ± 0.4	10.7 ± 0.4	F = 2.361, p = 0.095
BMI (mean ± std)				Independent samples test
2012–2013	16.5 ± 3.0	17.4 ± 3.6	16.5 ± 3.0	F = 1.443, p = 0.353
2013–2014	17.4 ± 3.8	17.4 ± 3.4	17.4 ± 3.6	F = 2.012, p = 0.874
2014–2015	18.5 ± 4.3	18.4 ± 3.8	18.4 ± 4.1	F = 2.798, p = 0.560
2015–2016	19.4 ± 4.2	19.4 ± 4.2	19.4 ± 4.2	F = 0.077, p = 0.706
DMFT^1^ (mean ± std)				Mann-Whitney test
2012–2013	0.05 ± 0.30	0.03 ± 0.22	0.04 ± 0.26	Z = –1.323 p = 0.186
2013–2014	0.15 ± 0.56	0.09 ± 0.36	0.12 ± 0.46	Z = –2.461 p = 0.014
2014–2015	0.27 ± 0.70	0.16 ± 0.51	0.21 ± 0.60	Z = –4.010 p = 0.000
2015–2016	0.37 ± 0.85	0.25 ± 0.65	0.31 ± 0.74	Z = –3.905 p = 0.000
DMFT (M_4_)^2^ ( mean ± std)*				Mann-Whitney test
2012–2013	0.04 ± 0.30	0.02 ± 0.19	0.03 ± 0.24	Z = –1.538 p = 0.124
2013–2014	0.14 ± 0.55	0.08 ± 0.34	0.11 ± 0.45	Z = –2.700 p = 0.007
2014–2015	0.26 ± 0.69	0.15 ± 0.49	0.20 ± 0.59	Z = –3.949 p = 0.000
2015–2016	0.34 ± 0.78	0.23 ± 0.60	0.28 ± 0.69	Z = –3.836 p = 0.000
DMFT (–M_4_)^3^ ( mean ± std)*				Mann-Whitney test
2012–2013	0.00 ± 0.05	0.01 ± 0.10	0.00 ± 0.08	Z = –0.551 p = 0.582
2013–2014	0.01 ± 0.12	0.01 ± 0.12	0.01 ± 0.12	Z = –0.486 p = 0.627
2014–2015	0.01 ± 0.13	0.01 ± 0.12	0.01 ± 0.12	Z = –0.107 p = 0.915
2015–2016	0.03 ± 0.26	0.03 ± 0.22	0.03 ± 0.24	Z = –0.830 p = 0.407

^1^: DMFT: decayed, missing, and filled teeth values for all permanent teeth.

2: DMFT (M4): decayed, missing, and filled teeth values only for the four first molars.

3: DMFT (M4): decayed, missing, and filled teeth values for permanent teeth except the four first molars.

### Determination of the Preventive Effect of the Sealant by Bivariate Analysis

The DMFT (M4) values in the sealant group and the no sealant group during the four annual check-up sections are shown in Table 1. No statistically significant difference was found between the sealant group and the no sealant group at baseline (0.02 vs 0.04, Mann–Whitney test, p >0.05). Compared with the DMFT (M4) values in the no sealant group, DMFT (M4) values in the sealant group were decreased significantly (Mann–Whitney test, p <0.05) by 44.1%, 40.2%, and 33.9%, respectively, during the following three annual check-up sections.

The prevalence of caries in the four first molars in the sealant group and the no sealant group during the four annual check-up sections are shown in Table 2. No statistically significant difference was found in the prevalence between the two groups at baseline (1.8% vs 2.6%, Chi-square test, p >0.05). Compared with the prevalence of caries in the four first molars in the no sealant group, in the four first molars in the sealant group this was decreased significantly (Chi-square test, p <0.05) by 29.8%, 30.5%, and 24.8%, respectively, during the following three annual check-up sections.

**Table 2:** Prevalence of caries in the four first molars* at the four annual check-up visits

Academic year	No sealant group	Sealant group	Total	Chi-square test (p)
	(n = 1325)	(n = 1648)	(n = 2973)	
	%	%	%	
2012–2013	2.6	1.8	2.2	0.132
2013–2014	7.9	5.3	6.5	0.007
2014–2015	11.8	8.2	9.8	0.001
2015–2016	15.4	11.7	13.3	0.003

** The percentage of children who had caries in the four first permanent molars.

Participants who had caries of the four first molars at baseline (n = 65) were excluded, and 2908 children were analysed for evaluating new caries during the observation period, as shown in Table 3. Caries incidences of the four first molars in the no sealant group after 1 year, 2 years, and 3 years were 5.9%, 13.1%, and 18.1%, respectively. The corresponding values in the sealant group were 4.2%, 9.1%, and 13.6%, respectively (Chi-square test of the sealant group vs the no sealant group, p <0.05). Compared with caries incidences in the no sealant group, caries incidences in the sealant group were decreased significantly (Chi-square test, p <0.05) by 28.7%, 30.7%, and 24.7%, respectively (Fig 2), during the following three annual check-up sections.

**Table 3:** Incidences of caries in the four first molars* at the four annual check-up visits (n = 2908)

Academic year	No sealant group	Sealant group	Total	Chi-square test (p)
	(n = 1290)	(n = 1618)	(n = 2908)	
	%	%	%	
2012–2013	0.0	0.0	0.0	–
2013–2014	5.9	4.2	5.0	0.039
2014–2015	13.1	9.1	10.9	0.004
2015–2016	18.1	13.6	15.6	0.028

* The percentage of children who developed new caries in the four first permanent molars.

### Binary Logistic Regression Analysis Results

The caries status of the four first molars during the last annual check-up section was recorded as a binary dependent variable (with/without caries). Participants who had caries of the four first molars at baseline (n = 65) were excluded, and 2908 children were analysed. The independent variables included gender, sealant group/no sealant group, primary caries status at baseline (with/without caries), age, and BMI at the last evaluation. BMI index was recorded as an ordinal variable (normal, overweight, and obese) considering age and gender.^9^ Binary logistic regression (enter method) was used to identify the associated variables, as shown in Table 4. Girls, and those who had dental caries in the primary dentition at baseline, were more likely to develop dental caries in their permanent molars during the study. The participants who received fissure sealants were less likely to develop caries, and the risk ratio was 0.73 (95% CI, 0.60–0.90; p <0.003).

**Table 4:** Binary logistic regression analysis for caries status of the four first molars at the last annual check-up section (with or without caries, n = 2908)

Variables	Dummy variables	B	SE	Wald	Sig	Exp(B) *	95% CI	
							Lower	Upper
Gender								
	Male							
	Female	0.4	0.11	14.16	0	1.5	1.21	1.84
Sealant								
	No sealant group							
	Sealant group	–0.31	0.1	8.99	0.003	0.73	0.6	0.9
Age at evaluation (years)								
	Continuous	0.23	0.12	3.69	0.055	1.26	0.99	1.59
Body height								
	Normal							
	Overweight	–0.09	0.16	0.36	0.551	0.91	0.67	1.24
	Obesity	–0.09	0.14	0.36	0.548	0.92	0.69	1.21
Primary caries at baseline								
	dmft = 0							
	dmft >0	0.8	0.12	43.07	0	2.21	1.75	2.81
Constant		–5.32	1.55	11.8	0.001	0.01		

* Exp(B) = odds ratio (OR).

## Discussion

The purpose of this longitudinal study was to evaluate the caries-preventive effect of the sealant public health programme that had been conducted in Beijing, the capital of China, showing low caries prevalence in permanent teeth. The prevalence and incidence of dental caries in permanent teeth among these participants were in accordance with the results of another investigation conducted in school children living in Beijing.^8^ The permanent teeth of girl students were more likely to decay than those of boy students, and this finding was in agreement with published studies.^11,13^ Therefore, the examinations for assessment of dental caries through these annual physical examinations were reliable, although critical calibration results were not available.

This was not a randomised controlled study. The classification of participants into the sealant group and the no sealant group was based on the record showing whether they were covered and sealed during this public health programme. The difference in dental caries status between the sealant group and the no sealant group at the evaluation section might have resulted from the intervention with the sealant or the different caries risk levels at baseline. General information and the prevalence of caries in both primary teeth and permanent teeth at baseline were balanced between the sealant group and the no sealant group. Therefore, the dental caries-preventive effect of this public health programme was confirmed. Based on the third and fourth national oral health epidemiology surveys conducted in 2005 and 2015, respectively, caries prevalence in permanent teeth among subjects living in Beijing ranged from 26.4% to 28.3%, which differed from the increasing national trend (from 28.9% to 38.5%).^22^ This difference might have occurred due to the benefit of this pit and fissure sealant public health programme. According to the 4th National Oral Health Epidemiology Survey conducted in 2015–2016, prevalence of dental caries and related factors, such as oral hygiene behaviour, sugar consumption habits, utilisation of dental services, and some aspects of oral health knowledge, were significantly different between 12-year-olds from regions with and without coverage of the national pit and fissure sealant programme in China.^22^ The national survey was a cross-sectional study, which could not confirm the causal relationship between the national sealant programme and dental caries status with certainty. However, it could provide a preliminary estimate for the assumption of benefits brought about by the sealant public health program.

The effectiveness of pit and fissure sealants in preventing tooth decay in permanent teeth was systematically reviewed by the Cochrane Collaboration.^1^ The risk ratio (95% CI) for resin-based sealant versus no treatment for caries yes/no at 24 months was 0.11 (95% CI, 0.06–0.22),^6,7,10^ which was much higher than the result obtained in this study, where the risk ratio was 0.73 (95% CI, 0.60–0.90) at 28 months. Three reasons might explain this difference. First, the effectiveness of sealants is obvious at a high caries risk level. All three studies were conducted in the 1970s, and the caries levels were much higher than the current situation in Beijing, China. Second, these three studies had a split-mouth design, and only the occlusion surface was assessed. The present research is a parallel-group study and the minimal analysis unit is the person: all the surfaces of the first molars were assessed, and a child with at least one sealed molar was categorised into the sealant group. Therefore, the sensitivity of the test index in the present study was relatively low. The risk ratio for resin-based sealant versus no treatment for caries yes/no at 24 months was 0.32 (95% CI, 0.13–0.82) in another randomised and parallel-group study 13 conducted in southern China. Caries prevalence was also low and in line with that in our study population; however, the caries-preventive effect was higher than that in our study. Third, the reason is that the organisation and implementation of a pit and fissure sealant in this dental public health programme differed from those in standard randomised controlled trials (RCTs). As a public programme in China, a country with a large population, the number of children who were covered was high, and more than 200 dental professionals were involved in this particular programme. Although these dental professionals had received standard training, the consistency of the clinical procedure could not be evaluated as critically as that in clinical trials. Also, the selection of indications may have been more inclusive and less consistent.

According to the meta-analysis result of randomised clinical trials,^17^ the pooled permanent tooth surfaces D(M)FS prevented fraction (PF) estimate comparing fluoride varnish with placebo or no treatment was 43% (95% confidence interval (CI) 30–57%; p <0.0001) in children and adolescents, and the PF was 28% (95% CI 19–36%) for fluoride gel^16^ and that for fluoride mouthrinse was 0.27 (95% CI 0.23–0.30).^15^ The PF was 27 % (95% CI 10% to 40%) for the current sealant public health programme, which was comparable with that of fluoride gel and fluoride mouthrinse. The sealant programme is resource-consuming in terms of professional manpower and time,^2^ and therefore, the result of the cost-benefit analysis for this in the context of being a public health programme might be questionable. More research is needed to determine the priority for a school-based sealant or fluoridation programme in the current circumstances in China.

In conclusion, the pit and fissure sealant public health programme conducted in Beijing was effective in preventing dental caries in the first permanent molars despite the low caries risk background. However, the caries-preventive effect of this public health programme was relatively low.
